# Secondary amyloidosis associated with heroin use and recurrent infections – A case report

**DOI:** 10.1016/j.amsu.2018.11.013

**Published:** 2018-11-22

**Authors:** Deepthi Mani

**Affiliations:** a Multi Care Good Samaritan Hospital, Puyallup, WA, USA; b University of Washington, Department of Family Medicine, Seattle, WA, USA

**Keywords:** Heroin use, Secondary amyloidosis, Renal amyloidosis

## Abstract

A 49-year-old lady with history of polysubstance use disorder, recurrent cutaneous abscesses, spinal diskitis and septic thrombophlebitis presented to the emergency room with complaints of intermittent fevers, worsening right hip pain and bilateral lower extremity edema. A month before the presentation, she had left another hospital against medical advice after being diagnosed with Methicillin-resistant *Staphylococcus aureus* bacteremia and right hip septic arthritis. Post discharge, she was off antibiotics, but continued heroin and methamphetamine use. On admission, she had right hip chronic osteomyelitis and was also in acute renal failure with evidence of nephrotic range proteinuria. Her renal biopsy subsequently revealed acute tubular necrosis and secondary (AA) amyloidosis with the classic apple green birefringence and positive immunohistochemical stain for serum amyloid A protein. Secondary amyloidosis, where there is deposition of fibrils composed of fragments of the acute phase reactant - serum amyloid A protein, often complicates chronic diseases with ongoing or recurring inflammation like spondyloarthropathies, inflammatory bowel disease and heredofamilial periodic fever syndromes.

Epidemiological studies now indicate that chronic inflammation as noted in illicit drug users, especially heroin users is on the rise as the etiology for AA amyloidosis in some parts of the developed world. The most common organ system involved in AA amyloidosis is the kidney. Given the opioid epidemic, clinicians are more likely to encounter similar cases of secondary amyloidosis.

## Highlights

• AA amyloidosis occurs in chronic inflammatory states due to deposition of fibrils of serum amyloid A protein fragments.
• Injection drug use and recurrent skin/soft tissue infections are associated with AA amyloidosis. The most common organ system involved in AA amyloidosis is the kidney.
• Renal amyloidosis should be considered as a potential cause for proteinuria/renal impairment in individuals who use heroin.

## 1 Introduction

Illicit drug use can result in multiple complications including drug overdoses, comorbid psychiatric disorders, HIV and hepatitis C virus infections, other infectious diseases including skin and soft tissue infections, endocarditis, and trauma. In the United States, opioid-involved overdoses accounted for two thirds of drug overdose deaths, with increases in multiple states across various age, racial/ethnic groups and urbanization levels [1]. Opioid use disorder can involve misuse of prescribed opioid medications, use of diverted opioid medications, or use of illicitly obtained heroin. There has been a significant increase in the rate of past-year heroin use in the United States between 2002–2004 and 2011–2013 from 1.6 per 1000 persons to 2.6 per 1000 persons [2]. The highest rates are noted among males, people aged 18–25 years, low income population, people living in urban areas, and in people with no health insurance or with Medicaid. However, the greatest increases in heroin use occurred in demographic groups that historically have had lower rates of heroin use, with rates doubling among women and more than doubling among non-Hispanic whites.

Here in we present the clinical case of a woman with active heroin and methamphetamine use who presented with sepsis from osteomyelitis and a less recognized complication of injection drug use - AA amyloidosis. She consented to have the case report published in a scientific journal.

## 2 Case presentation

**Fig. 1:**
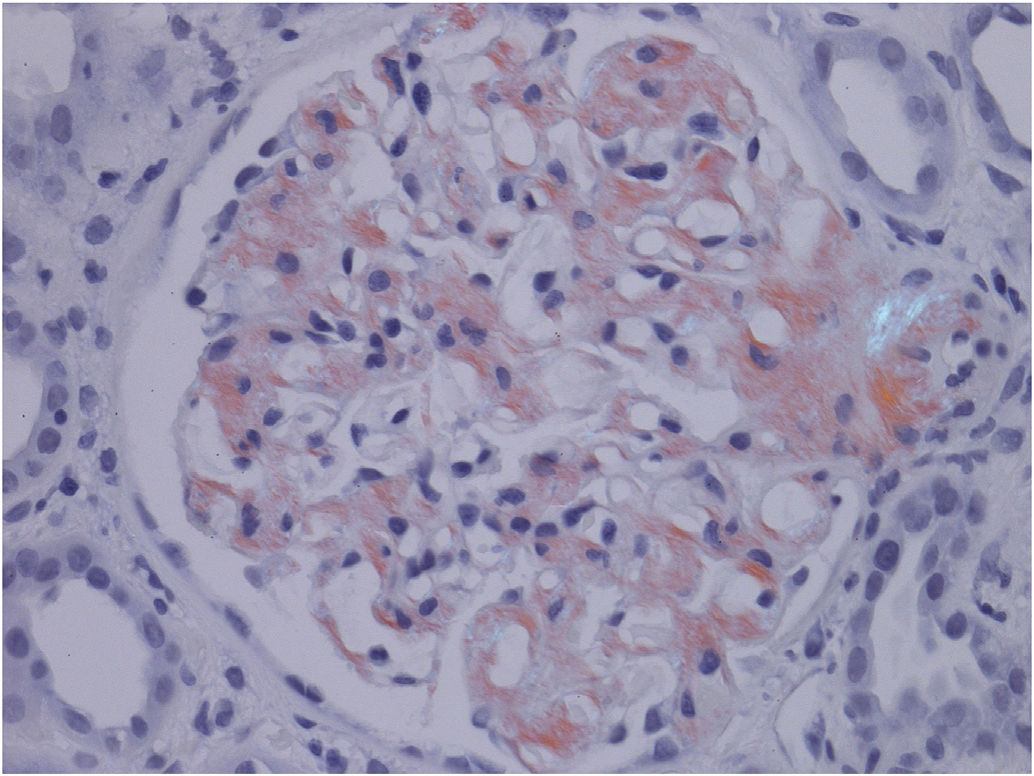
Amyloid deposits in the glomeruli stained red with Congo red show the characteristic apple green birefringence under polarized microscopy. (For interpretation of the references to colour in this figure legend, the reader is referred to the Web version of this article.)

**Fig. 2:**
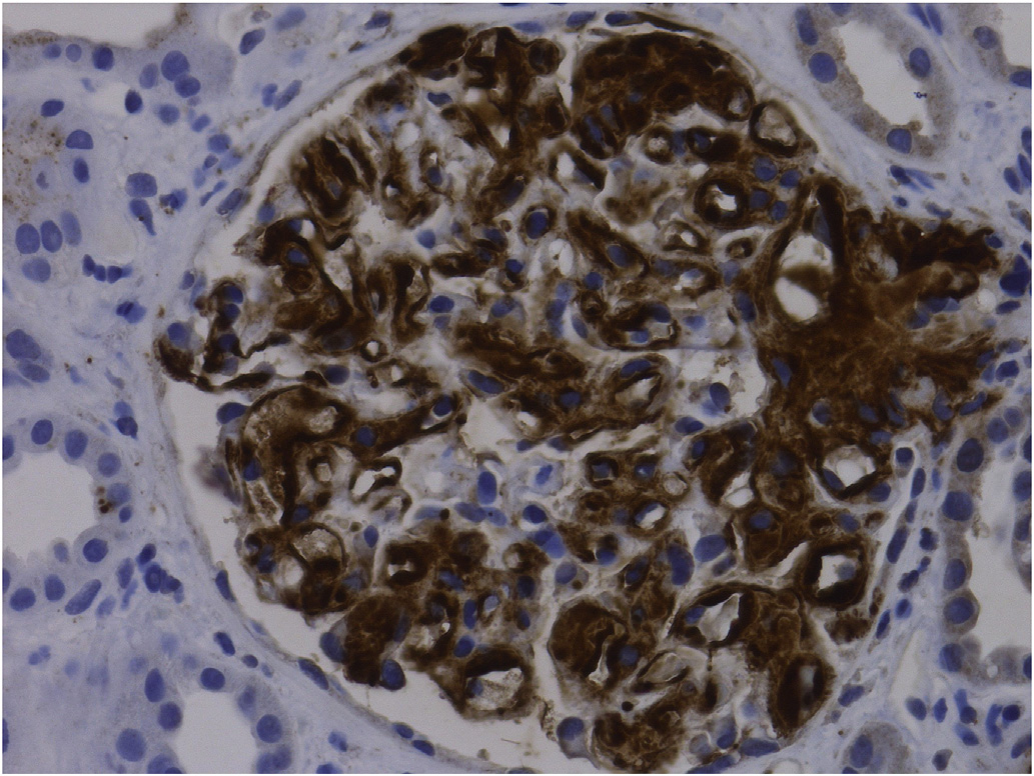
Positive immunohistochemical stain for serum amyloid A protein.

A 49-year-old Caucasian lady with history of polysubstance use disorder and related complications including, recurrent cutaneous abscesses, spinal diskitis and septic thrombophlebitis presented to the emergency room with complaints of intermittent fevers and right hip pain. A month prior to the presentation, she had left another hospital against medical advice after being diagnosed with Methicillin-resistant *Staphylococcus aureus* bacteremia and right hip septic arthritis. Post discharge, she was off antibiotics, but continued heroin and methamphetamine and was taking multiple dose of ibuprofen in addition for pain control. On admission, her physical exam was notable for severe tenderness in her right hip, marked bilateral lower extremity edema and multiple deep, circular, punched-out looking atrophic scars involving all extremities at prior skin popping (subcutaneous injection of illicit drugs) sites. Pertinent laboratory data included chronic anemia with a hemoglobin of 9.8 g/dL, WBC count of 10.23 k/uL and a platelet count of 395 k/uL. She had negative HIV, Hepatitis B antibodies and elevated Hepatitis C antibody with undetectable Hepatitis C viral load. Her basic metabolic profile noted a sodium of 140 mmol/L, potassium of 3.5 mmol/L and a creatinine of 2.9 mg/dL (estimated glomerular filtration rate of 17 ml/min) which was a significant rise from the normal creatinine levels a month earlier. Her urine analysis noted >500mg/dL proteinuria with a protein/creatinine ratio of 28.25. She had no monoclonal proteins on serum or urine electrophoresis. CT scan of her right hip noted marked degenerative changes in the right hip, with right acetabular protrusion and cortical disruption of the medial acetabular wall. She was diagnosed with right hip osteomyelitis and was in acute renal failure with evidence of nephrotic range proteinuria. She was placed on antibiotics (daptomycin) and underwent hip arthroscopy with irrigation and debridement along with lavage shortly after admission. Differential diagnoses considered for her renal disease included acute tubular necrosis due to sepsis, post infectious glomerulonephritis, focal segmental glomerulosclerosis associated with heroin use, acute interstitial nephritis from NSAIDs and membranoproliferative glomerulonephritis associated with Hepatitis C. She underwent a renal biopsy which revealed acute tubular necrosis and secondary (AA) amyloidosis with the classic apple green birefringence when stained with Congo red (Fig. 1) and positive immunohistochemical stain for serum amyloid A protein (Fig. 2). Two weeks after admission she underwent Girdlestone arthroplasty. During the hospital stay, she developed intermittent hypotension, had evidence of primary adrenal insufficiency attributed to amyloidosis and was initiated on steroids. She was discharged home after completion of prolonged antibiotic therapy with daptomycin and was maintained on oral doxycycline. She was referred to outpatient opioid treatment program. Eight months after her admission, she remained committed to be off all illicit drugs and underwent right total hip replacement. Her creatine levels normalized (estimated GFR of 82 ml/min) but she continued to have proteinuria from renal amyloidosis (protein/creatinine ratio of 28.25) and required diuretic therapy for symptomatic management of her edema.

## 3 Discussion

Amyloidosis is a generic term to describe disorders where there is extracellular deposition of fibrils composed of low molecular weight protein subunits of a variety of proteins. These subunits are derived from soluble precursors which undergo conformational changes that lead to the adoption of a predominantly antiparallel beta-pleated sheet configuration. Two major forms of amyloidosis are AL amyloidosis and AA amyloidosis. Other forms of amyloidosis seen clinically include dialysis-related amyloidosis, heritable amyloidoses, age-related systemic amyloidosis and organ-specific amyloidosis. AL amyloidosis is a systemic disorder where a plasma cell dyscrasia results in deposition of protein derived from immunoglobulin light chain fragments. AA amyloidosis (secondary amyloidosis) occurs when there is deposition of fibrils composed of fragments of the acute phase reactant - serum amyloid A protein (SAA), which is an apolipoprotein constituent of high-density lipoprotein that is synthesized by hepatocytes under the transcriptional regulation of proinflammatory cytokines like IL-1, IL-6 and TNF-alpha [3,4]. Sustained overproduction of SAA is a prerequisite for the development of AA amyloidosis, Patients with chronic diseases with ongoing or recurring inflammation like rheumatoid arthritis (RA), spondyloarthropathies, inflammatory bowel disease, chronic infections like tuberculosis, bronchiectasis, osteomyelitis and heredofamilial periodic fever syndromes like familial Mediterranean fever are at risk of developing AA amyloidosis. Autoimmune conditions including RA were thought to be common etiologic agents in the developed world whereas untreated chronic infections were considered the predominant cause in countries with limited medical resources.

Epidemiological studies now indicate that chronic inflammation as noted in illicit drug users, especially heroin users is on the rise as the etiology for AA amyloidosis in some parts of the developed world [5,6]. In a retrospective study of 625 patients who attended a single national center in United Kingdom spanning 25 years, juvenile idiopathic arthritis and RA were no longer the main causative factors of AA amyloidosis in patients in the 2007–2014 cohort when compared to earlier cohorts, most likely related to the emergence of effective treatment strategies including biologics for these disorders. Greater proportions of recent AA patients had uncharacterized underlying inflammatory disorders or chronic infection secondary to injection drug use. Injection drug use was the etiologic agent in 1% in the 1990–1997 cohort of AA amyloidosis patients, compared to 13% in the 2007–2014 cohort [6]. In a case series of 24 patients with biopsy proven renal amyloidosis, from 1998 to 2013 in a county hospital in San Francisco, all 24 had a history of injection drug use [7]. In a case control study from two large urban medical centers in Seattle, 38 patients who had biopsy proven renal amyloidosis in the 2005–2015 timeframe, 95% had a prior history of heroin use, 87% had skin abscesses, and 76% had evidence of muscling and 27% had history of skin popping the drug. Chronic autoimmune disorders were uncommon among case patients in this study. Among the case group, 6 patients had a past history of tuberculosis, 4 were HIV positive, 2 had systemic lupus, 1 had rheumatoid arthritis, 1 had inflammatory bowel disease and 1 had a serum monoclonal protein confirmed by serum and urine immunofixation [8]. Retrospective studies from Norway and Germany noted heroin use in 50–70% of the patients diagnosed with heroin use in various study periods during the past 15 years [9,10].

The most likely mechanism linking injection heroin use with AA amyloidosis in this population is the development of recurrent soft-tissue infections with trigger hepatic production of serum amyloid A protein. Suppurative infections are common among injection drug users where there is of widespread availability of black tar heroin, which contains impurities that promote venous sclerosis and use of secondary muscle and skin injection sites. The majority of patients in the case control study from Seattle had documentation of muscling or skin-popping, and most had evidence of skin abscesses as noted in the current patient, though generalizability of the findings could be limited by the low and varying incidence of AA amyloidosis noted within different geographic locations [8].

AA amyloidosis predominantly presents with renal involvement. Proteinuria leading to nephrotic syndrome and renal insufficiency are the earliest and most frequent clinical manifestation that should raise suspicion of AA amyloidosis in patients with chronic inflammatory conditions [3,4]. Hepatosplenomegaly and adrenal insufficiency may complicate the disease course. Gastrointestinal tract may also be affected, causing malabsorption, intestinal pseudo-obstruction, diarrhea, or bleeding. Peripheral polyneuropathy, restrictive myocardiopathy leading to heart failure, and skin and soft tissue involvement, such as macroglossia, are extremely uncommon, especially when compared with other types of systemic amyloidosis like AL amyloidosis [3,4].

Diagnosis of AA amyloidosis is based on clinical organ involvement and histological demonstration of amyloid deposits. The amyloid deposits appear as amorphous hyaline material on light microscopy. The fibrils bind Congo red leading to green birefringence under polarized light. The diagnosis of AA amyloidosis can be confirmed by positive immunoperoxidase staining of deposits using monocloncal antibody against AA amyloid protein. Scintigraphy with radioisotope labeled serum amyloid P component, though not widely available, is a useful screening technique for suspected amyloidosis. Once AA amyloid is present, long-term suppression of the circulating SAA level is vital in improving outcomes through aggressively treating the underlying disease with the aim of persistent normalization of the hepatic acute-phase response. Treatment includes antibiotics, disease modifying agents and biologics based on etiology of inflammation, together with supportive and symptomatic care [3,4]. Patients with renal amyloidosis who progress to end-stage renal disease can be treated with either dialysis or renal transplantation. Diuretics are often required, especially high doses of loop diuretics in combination with either thiazide or potassium-sparing diuretics. Angiotensin-converting enzyme inhibitors reduce the risk of progression to ESRD and proteinuria, but whether these confer any long-term benefit in AA amyloidosis specifically is unknown [11]. In patients with AA amyloidosis where the underlying condition is refractory to treatment, development of targeted therapies to inhibit amyloid formation and enhance the clearance of existing deposits are currently being studied [12].

## 4 Conclusion

Injection drug use and recurrent infections can result in AA amyloidosis which presents primarily with renal disease and nephrotic range proteinuria. Given the opioid epidemic and the rising heroin use, clinicians are more likely to encounter similar cases of secondary amyloidosis in their practice. Public health policies to prevent and respond to the epidemic through safe prescribing practices, harm reduction strategies, easy access and availability to treatment including medication-assisted treatment, law enforcement strategies to reduce the illicit drug supply all could help curb the complications from illicit drug use including recurrent infections and secondary amyloidosis.

## Ethical approval

The patient consented to have the case report published in a scientific journal.

## Sources of funding

None.

## Author contribution

Single author article.

## Conflicts of interest

None.

## Research registration number

None.

## Guarantor

Deepthi Mani MD (Single author article).

## Provenance and peer review

Not commissioned, externally peer reviewed.

## Appendix A Supplementary data

CAREchecklist (1) CAREchecklist (1)
